# Editorial Note: HIV gp120 Induces Mucus Formation in Human Bronchial Epithelial Cells through CXCR4/α7-Nicotinic Acetylcholine Receptors

**DOI:** 10.1371/journal.pone.0294613

**Published:** 2023-11-14

**Authors:** The PLOS ONE Editors

After this article [1] was published, concerns were raised that the SIV panel in Fig 4D partially overlaps with the SIV+ART panel in Fig 5A, when rotated 180 degrees.

During editorial follow up on this issue, the corresponding author stated that the data were correctly reported in Fig 4D, and the Fig 4D SIV panel was erroneously used to represent the Fig 5A SIV+ART panel. The corresponding author also stated that an updated panel cannot be provided and that none of the raw data underlying the article’s results are currently available.

An Academic Editor reviewed the concerns and stated that the article’s overall results and conclusions [1] remain supported by other results reported in the article, including the cell culture data and the data obtained from HIV-patients.
